# Draft Genome Sequences of *Burkholderia contaminans*, a *Burkholderia cepacia* Complex Species That Is Increasingly Recovered from Cystic Fibrosis Patients

**DOI:** 10.1128/genomeA.00766-15

**Published:** 2015-08-06

**Authors:** Ruhi A. M. Bloodworth, Carrie Selin, Maria Agustina López De Volder, Pavel Drevinek, Laura Galanternik, José Degrossi, Silvia T. Cardona

**Affiliations:** a Department of Microbiology, University of Manitoba, Winnipeg, Canada; b Facultad de Farmacia y Bioquímica, Universidad de Buenos Aires, Buenos Aires, Argentina; c Department of Medical Microbiology, 2nd Faculty of Medicine, Charles University and Motol University Hospital, Prague, Czech Republic; d Hospital de Niños Ricardo Gutierrez, Buenos Aires, Argentina; e Department of Medical Microbiology & Infectious Disease, University of Manitoba, Winnipeg, Canada

## Abstract

*Burkholderia contaminans* belongs to the *Burkholderia cepacia* complex (BCC), a group of bacteria that are ubiquitous in the environment and capable of infecting the immunocompromised and people with cystic fibrosis. We report here draft genome sequences for the *B. contaminans* type strain LMG 23361 and an Argentinian cystic fibrosis sputum isolate.

## GENOME ANNOUNCEMENT

*Burkholderia contaminans* (1) is a species of the *Burkholderia cepacia* complex (BCC), a group of at least 17 species that infect immunocompromised individuals, in particular those with cystic fibrosis (CF) (2, 3). While *B. cenocepacia* and *Burkholderia multivorans* are more prevalent in CF patients in the United States and Canada (4), *B. contaminans* is highly represented in Argentina and Portugal (5, 6), and its incidence is increasing in Spain (7). *B. contaminans* (1) received its species name in reference to the contamination of a Sargasso Sea DNA sample (8) with the so-called *Burkholderia* SAR-1 metagenome (9). Intriguingly, *B. contaminans* has also been found as a contaminant of pharmaceutical products (10, 11). Thus, genome sequence analysis is expected to shed light on whether *B. contaminans* has an enhanced capacity to survive in harsh environments in comparison with other BCC species that are also isolated from man-made products (12). Here, we used single-molecule real-time (SMRT) sequencing to sequence the genomes of *B. contaminans* LMG 23361 (1), the type strain for the species*,* and *B. contaminans* FFH2055, an isolate from the sputum from a CF patient in Buenos Aires, Argentina.

Cultures were grown in LB, and genomic DNA was isolated using phenol-chloroform, as per Sambrook and Russell (13). Sequencing-ready libraries were prepared at the Duke University Genome Sequencing & Analysis Core Resource. DNA sequencing was performed using the PacBio RS II system and yielded 237,907 reads with a mean length of 8.7 kb for LMG23361, and 256,171 reads with a mean read length of 7 kb for FFH2055. The reads were assembled using HGAP (14) (PacBio SMRT Analysis software version 2.3), followed by polishing using Quiver (PacBio). The assembly of LMG23361 consisted of 17 contigs containing 9.2 Mb of sequence, while the FFH2055 assembly contained 8.2 Mb organized into 8 contigs. Species in the genus *Burkholderia* are known for having large multipart genomes, and the sizes of our assemblies fell within the range of 7.4 to 9.73 Mb seen in previously sequenced genomes (15). Annotation of the assemblies with RAST (16) identified 8,674 and 7,641 open reading frames in LMG23361 and FFH2055, respectively, which fall within the range previously seen in BCC species. LMG23361 contained the complete core genome conserved across the order *Burkholderiales* (17), while FFH2055 was missing 8 conserved genes.

To our knowledge, the draft genome sequence of FFH2055 is the first produced for a *B. contaminans* strain isolated from a cystic fibrosis patient. This provides a starting point for investigating the emerging prevalence of this new BCC pathogen.

### Nucleotide sequence accession numbers.

The *B. contaminans* LMG23361 and *B. contaminans* FFH2055 draft genomes have been deposited at DDBJ/EMBL/GenBank under the accession numbers LASD00000000 and LASC00000000, respectively. The versions described in this paper are the first versions, LASD01000000 and LASC01000000.
